# From de‐skilling to up‐skilling: How artificial intelligence will augment the modern physician

**DOI:** 10.1002/jeo2.70677

**Published:** 2026-03-03

**Authors:** Felix C. Oettl, James Pruneski, Balint Zsidai, Yinan Yu, David Fendrich, Thomas Tischer, Michael T. Hirschmann, Stefano Zaffagnini, Kristian Samuelsson, ESSKA Artificial Intelligence Working Group

**Affiliations:** ^1^ Department of Orthopedic Surgery Balgrist University Hospital, University of Zürich Zurich Switzerland; ^2^ Department of Orthopaedic Surgery Tripler Army Medical Center Honolulu Hawaii USA; ^3^ Sahlgrenska Sports Medicine Center Gothenburg Sweden; ^4^ Department of Orthopaedics Institute of Clinical Sciences, Sahlgrenska Academy University of Gothenburg Gothenburg Sweden; ^5^ Department of Orthopedics Skåne University Hospital Malmö/Lund Sweden; ^6^ Department of Computer Science and Engineering Chalmers University of Technology Gothenburg Sweden; ^7^ Tenfifty Gothenburg Sweden; ^8^ Department of Orthopaedic Surgery University Medicine Rostock Rostock Germany; ^9^ Department of Orthopaedic Surgery and Traumatology Kantonsspital Baselland Bruderholz Switzerland; ^10^ University of Basel Basel Switzerland; ^11^ Dipartimento di Scienze Biomediche e Neuromotorie (DIBINEM) University of Bologna Bologna Italy; ^12^ II Clinica Ortopedica e Traumatologica, IRCCS Istituto Ortopedico Rizzoli Bologna Italy; ^13^ Department for Orthopaedics Sahlgrenska University Hospital Mölndal Sweden

**Keywords:** artificial intelligence, augmentation, deskilling, medical education, orthopaedics, upskilling

## Abstract

The integration of artificial intelligence (AI) into orthopaedic practice is no longer a theoretical future but an inevitable reality. As AI models increasingly demonstrate superior performance in specific diagnostic and administrative tasks, concerns have arisen regarding the potential replacement of physicians and the erosion of clinical competency. This narrative review synthesizes current evidence to reframe the debate from a fear of replacement to a strategy of augmentation. Pathways leading to ‘deskilling’—the loss of existing expertise—and the emerging threat of ‘never‐skilling’, where trainees fail to acquire foundational proficiencies due to premature reliance on automation, are analysed. Current AI applications function primarily as assistants rather than autonomous agents, offering an opportunity for ‘upskilling’ by liberating clinicians from repetitive administrative burdens and standardizing diagnostic accuracy. However, realizing this benefit requires deliberate educational mechanisms; one has to argue that maintaining clinical excellence requires a shift in training paradigms, emphasizing critical oversight where human reasoning validates AI outputs. AI will not replace the orthopaedic surgeon in the foreseeable future; rather, it will necessitate an evolution of the physician's role. By automating routine tasks, AI allows the modern physician to operate at a higher level, focusing on complex decision‐making, procedural excellence and patient empathy. The future requires mechanisms to ensure AI remains a tool for professional elevation rather than a catalyst for skill degradation.

**Level of Evidence:**

Level V.

**Abbreviations**
AI: artificial intelligence
DAX: Dragon Ambient eXperience

## FRAMING THE REAL DEBATE—AUGMENTATION NOW, AUTOMATION LATER

**Figure 1:**
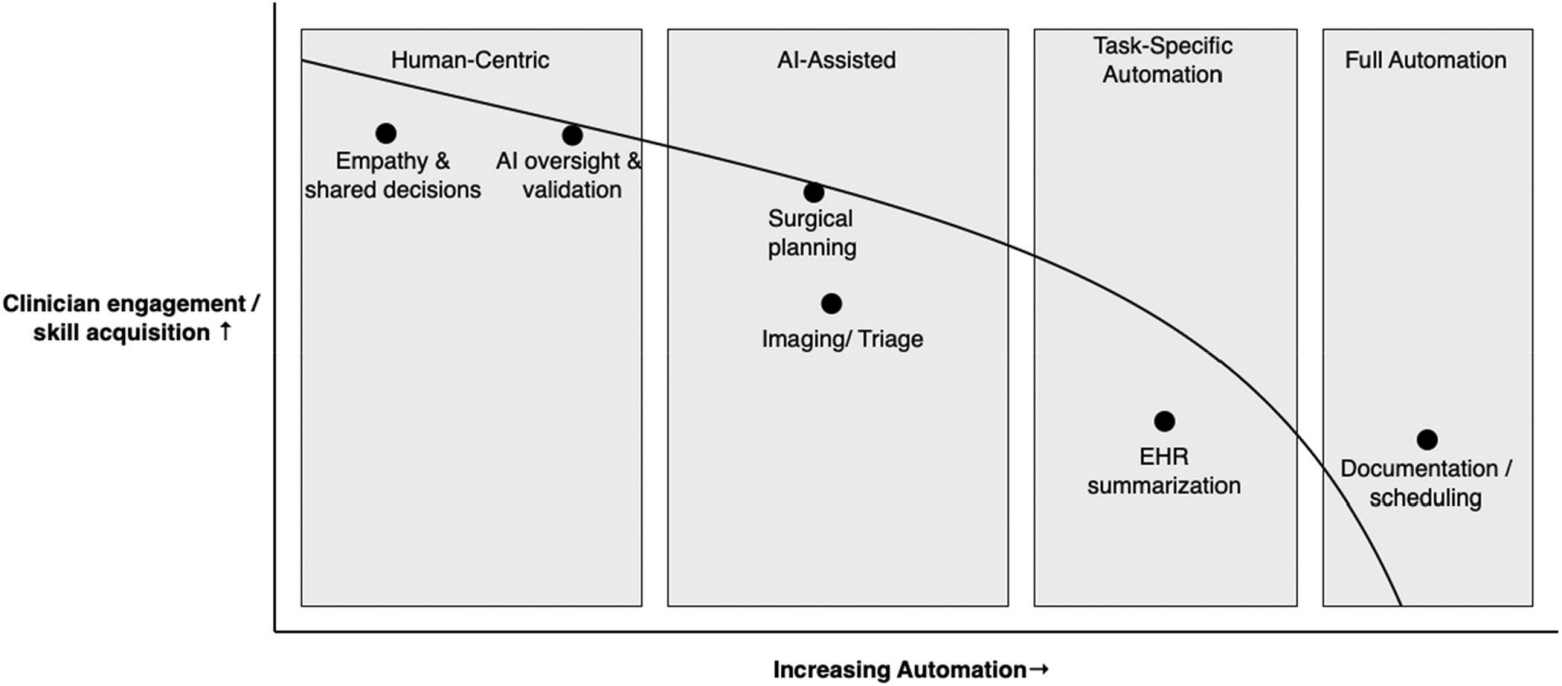
The augmentation–automation spectrum. This conceptual map situates common clinical tasks along a continuum from human‐centric practice to task‐specific and full automation. Current medical AI predominantly operates in the augmentation zone (Human Centric, AI‐Assisted), where clinician engagement and skill acquisition remain high for activities such as diagnostic interpretation, surgical planning and oversight, while routine administrative tasks migrate toward automation. AI, artificial intelligence; EHR, electronic health record.

There are an increasing number of publications highlighting the superior performance of artificial intelligence (AI) models and clinicians assisted by AI models compared to clinicians alone [13, 37, 55, 68]. These developments have raised important questions regarding how AI exposure may influence skill development, particularly for trainees who depend on hands‐on experience to acquire clinical proficiency. AI‐assisted workflows may help optimize and automate repetitive and resource‐intensive tasks, such as documentation, billing and patient scheduling (Figure 1) [17, 33]. While several studies report the enhanced performance of clinicians assisted by AI, overreliance on AI‐assisted workflows has been proposed to lead to a gradual decline in professional skills, clinical autonomy and decision‐making, referred to as ‘deskilling’ [2, 15, 38]. Overreliance on AI may be especially harmful for early‐career physicians, who fail to acquire essential clinical skills through experiential learning [1, 52].

The risk of never‐skilling—failure to develop essential competencies—represents a distinct and potentially more insidious threat than deskilling in the AI‐augmented clinical learning environment. Unlike deskilling, which describes the erosion of previously acquired skills through disuse, never‐skilling occurs when AI systems are introduced so early in a trainee's development that foundational clinical reasoning and procedural competencies are never adequately acquired in the first place [1].

Recent randomized controlled trials have begun to quantify the actual impact of AI‐assisted documentation tools on clinical workflows and physician burden. In a pragmatic three‐group trial involving 238 outpatient physicians across 14 specialties, two ambient AI scribe applications—Microsoft Dragon Ambient eXperience (DAX) Copilot and Nabla—were compared against usual care [32]. While Nabla users experienced a 9.5% reduction in time‐in‐note compared to controls, DAX users showed no statistically significant change in documentation time [32]. Importantly, both AI scribe groups demonstrated improvements in burnout‐related measures, including increased Mini‐Z scores and reduced physician task load and work exhaustion scores, yet these secondary endpoints suggest incremental rather than transformative gains in daily practice [32]. These findings highlight the distinction between documentation efficiency in optimized settings versus typical clinical implementation, underscoring that current‐generation AI scribes represent a partial solution to the documentation burden rather than a comprehensive resolution [28].

The increasing presence of AI in healthcare will have a far‐reaching impact on clinicians in orthopaedics eventually [41, 43, 44, 51, 56, 57]. Arguments can be made for both a positive and negative outlook regarding the long‐term impact of AI in the day‐to‐day clinical environment.

Key definitions

- *Deskilling*: Measurable decline in diagnostic, procedural or decision‐making ability due to reduced practice or overreliance on automated systems.
- *Upskilling*: Improvement in clinical competence or acquisition of higher‐order capabilities facilitated by interaction with AI tools.
- *Never‐skilling*: AI systems are introduced so early in a trainee's development that foundational clinical reasoning and procedural competencies are never adequately acquired in the first place.
- *Augmentation*: AI functioning as an assistant providing recommendations while clinicians retain full responsibility for interpretation and action.
- *Full automation*: AI executing a medical task independently without ongoing clinician oversight—a capability not yet applicable to orthopaedic workflows.

This narrative review synthesizes current evidence on the impact of AI‐assisted workflows on clinical competence and acquisition of competence in orthopaedics and describes where AI may support upskilling versus where the risk of deskilling persists.

## THE CURRENT ERA: AI AS AN ASSISTANT—UP‐SKILLING BY DESIGN

### Micro‐learning at the point of care

**Figure 2:**
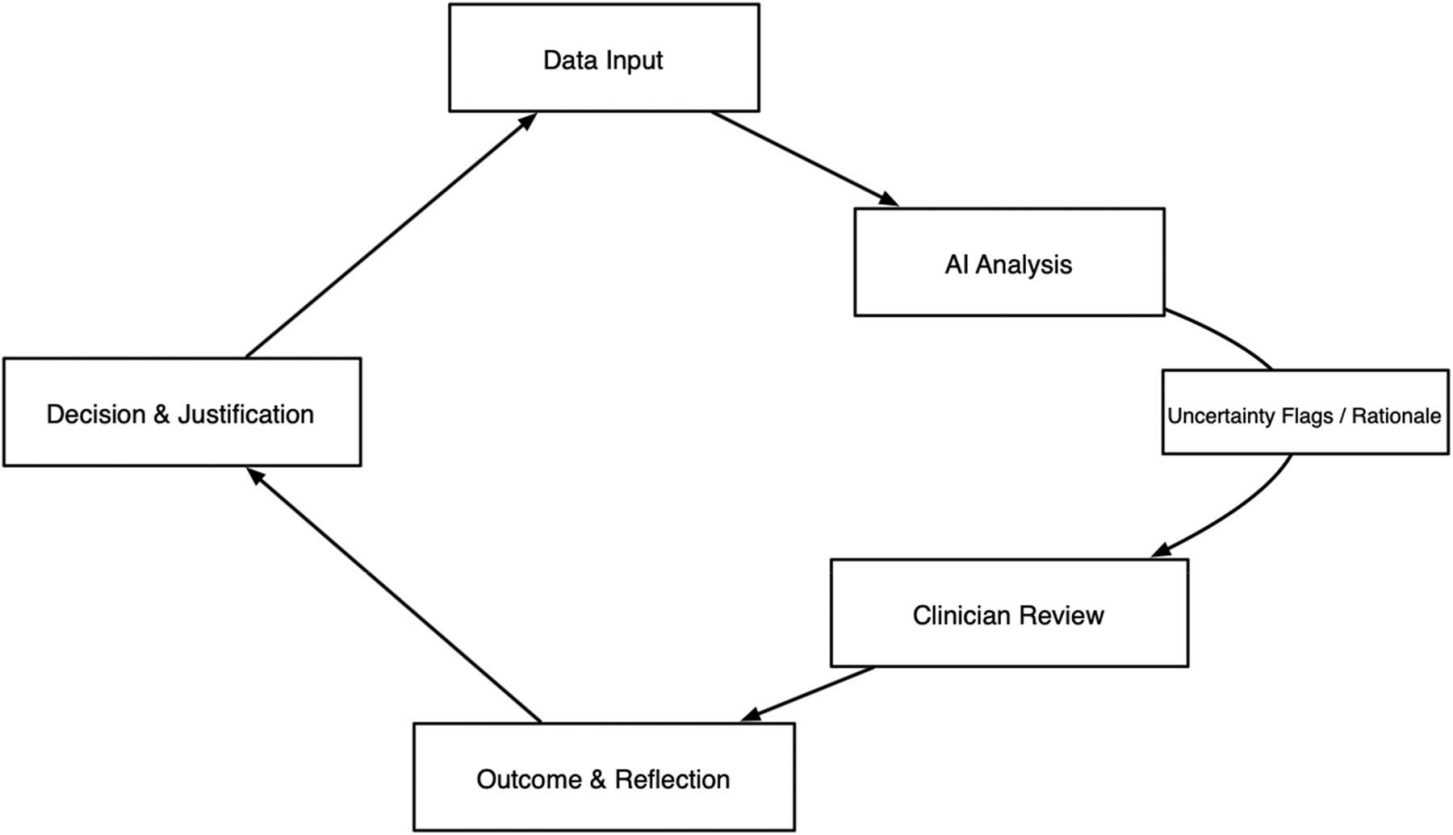
Human‐in‐the‐loop micro‐learning cycle. AI suggestions enter a closed loop of clinician inquiry, review and outcome‐based reflection, generating continuous micro‐learning at the point of care. By explicitly routing uncertainty and feedback into each cycle, the workflow reinforces expert reasoning and mitigates automation complacency. AI, artificial intelligence.

Currently, AI models serve as adjuncts for point‐of‐care processes and can assist with identification, classification and prognosis [27, 36, 44, 47]. This facilitates continuous micro‐learning: AI‐generated suggestions require clinicians to review, confirm or override recommendations, thus reinforcing diagnostic reasoning while avoiding passive reliance (Figure 2).

The supervision of AI use in clinical learning environments presents distinct challenges that extend beyond the simple restriction of AI tools. Recent educational frameworks have identified specific behavioural patterns—characterized as ‘cyborg’ and ‘centaur’ approaches—that clinicians adopt when engaging with AI, with cyborg practice involving tight intertwining of user and AI for each task, whereas centaur practice maintains division of tasks with preserved critical oversight [1]. Critically, educators themselves may lack sufficient familiarity with AI technologies that learners employ, creating an inversion of expertise where supervisors must simultaneously teach, learn and practice novel workflows [1]. This pedagogical challenge necessitates explicit teaching of critical thinking as a foundational competency, including structured recognition of moments when clinicians encounter AI‐generated outputs they cannot fully retrace—what the authors describe as ‘leaps of faith’ that demand pausing and deliberate evaluation [1]. Without formal integration of critical thinking frameworks during AI interaction, trainees risk developing automated reliance patterns that may generalize to complex clinical scenarios where independent reasoning remains essential, highlighting the necessity of intentional supervisory strategies that promote adaptive practice regardless of technology adoption.

Emerging evidence supports this learning benefit. For example, a recent study integrated an AI tool for lung disease scoring into radiology residents' workflow and found that residents who used the AI made significantly fewer scoring errors and achieved 22% higher inter‐rater agreement on chest X‐ray assessments [52]. Importantly, the residents remained vigilant to AI mistakes and rated the tool as highly useful and trustworthy. This demonstrates that, when AI is well‐integrated into clinical workflow, it can ‘upskill’ trainees by improving diagnostic accuracy and consistency while avoiding automation complacency.

### Liberating time for core clinical skills

Administrative burden represents a major barrier to maintaining clinical expertise [5, 10, 25, 46]. Over 74% of physicians identify clinical documentation as a primary contributor to burnout [14, 50]. AI‐driven ambient intelligence solutions reduce documentation time and after‐hours workload [4, 9, 20, 59].

This translates directly into reclaimed cognitive bandwidth and time enabling physicians to refocus on patient interaction, critical reasoning and procedural skill, the very foundations of clinical acumen. Moreover, recent integration of large language models into electronic health record systems has shown that AI can now approach or surpass human performance in summarizing and transcribing complex clinical narratives [22, 61, 70], as well as predicting future events [58, 65], marking a pivotal shift toward technology that enhances, rather than erodes, the practice of medicine.

### Raising the performance floor

AI‐assisted analyses of musculoskeletal imaging are of growing interest to the field, and AI tools have been shown to improve human performance in these diagnostic tasks [21, 24, 42]. These tools may standardize quality and reduce practice variation, representing a powerful, system‐wide form of up‐skilling. Beyond fracture detection, AI assistance may also improve performance in non‐binary grading and classification tasks, while improving interobserver agreement [12]. In general, performance improvements are predominantly observed in junior physicians and learners [12, 62], further highlighting the utility of AI for both raising performance and facilitating learning. For surgical tasks, AI‐assisted planning may improve precision and decrease operative times; however, further studies are required to characterize the impact of AI and robot‐assistance on long‐term patient outcomes [29, 31].

## THE FUTURE HORIZON: FULL AUTOMATION AND THE INEVITABLE SKILL SHIFT

### When de‐skilling becomes relevant

The current discourse around AI‐induced de‐skilling in healthcare conflates two fundamentally different technological phases. De‐skilling concerns become legitimate only when specific medical tasks achieve complete automation—a threshold we are far from reaching. Current AI applications in healthcare function primarily as augmentation tools, operating within narrowly defined parameters that require continuous human oversight [40, 42, 44, 49, 67].

### The ‘calculator’ precedent

Historical precedents of technological adoption provide valuable insights into the skill evolution process [48, 66]. The introduction of calculators in mathematics education offers a valuable parallel. Rather than diminishing capability, calculators enabled mathematicians to focus on higher‐order thinking, complex problem‐solving and advanced concepts. This technological shift exemplified a broader pattern observed across professional domains: automation of routine tasks facilitates upward skill migration toward more sophisticated competencies [7, 11, 66].

The literature consistently demonstrates that technological advancement leads to adaptation to technology, transformations and retraining of skills throughout the work life, rather than wholesale professional elimination [19, 26]. The talents and abilities safest from automation are those that are related to non‐linear abstract thinking—the kind of reasoning that characterizes expert physicians [7, 11, 19, 26, 66].

### The skill shift: Evolution, not elimination

**Figure 3:**
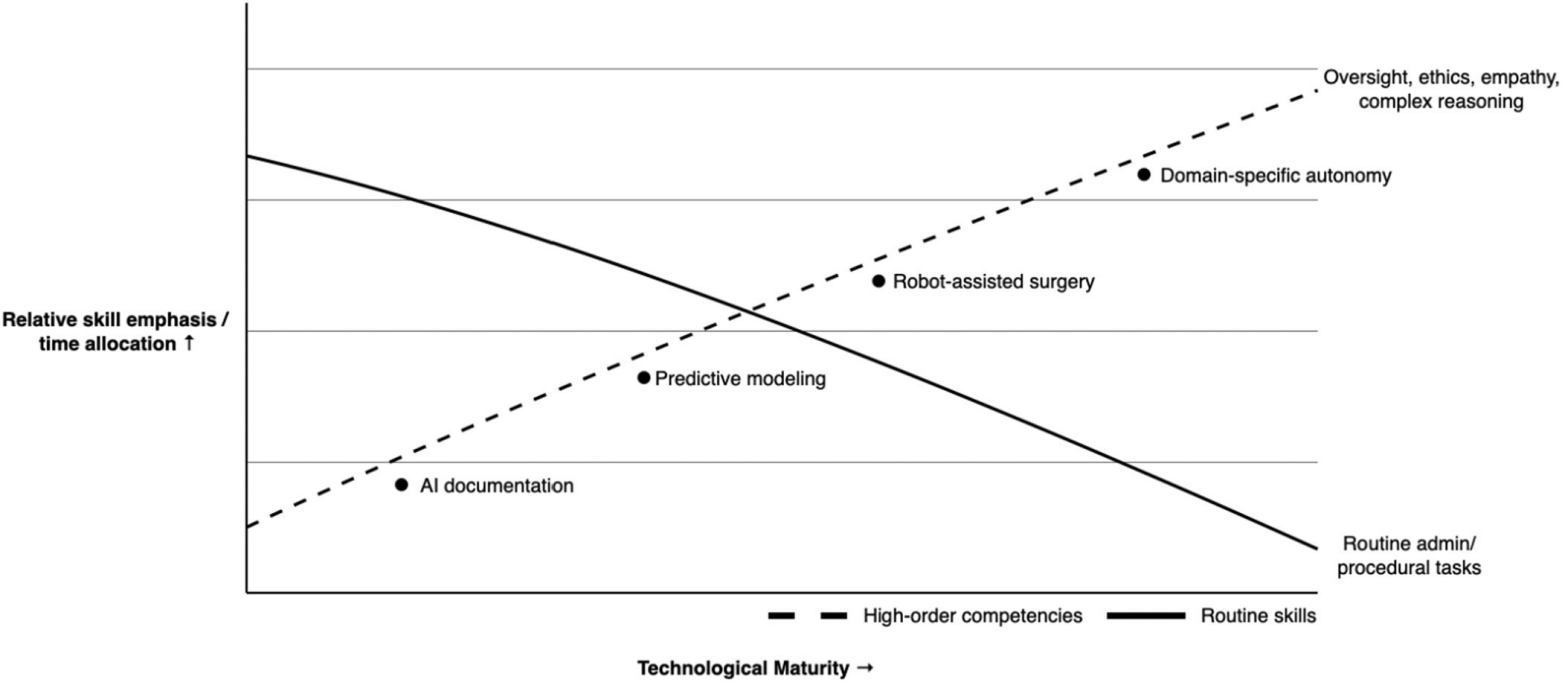
The skill evolution curve. As technology advances, routine administrative/procedural effort declines while the relative emphasis on high‐order competencies—oversight, ethics, empathy and complex clinical reasoning—rises. The trajectory reflects a shift in skill focus, rather than erosion, positioning physicians to operate at higher‐value layers of care as selective automation matures.

As medical AI systems eventually achieve full automation in specific domains, the physician's role will naturally evolve toward three critical areas that represent the profession's unique value proposition (Figure 3):

- *Managing and validating AI systems*: Training in AI oversight, such as technical use (how to use this instrument), medical decision making (when to use it) and critical assessment (should I use this tool?), will be necessary for future physicians [30, 34, 67]. The complexity of these responsibilities demands deep medical knowledge combined with understanding of model limitations and failure modes.
- *Deepening uniquely human skills*: The automation of routine tasks will enable physicians to concentrate on irreplaceable human competencies. These include complex procedural expertise that requires intervention from human experts, patient empathy and communication skills that research shows remain important in an AI‐enhanced healthcare system and ethical oversight responsibilities that ensure AI applications align with patient values and clinical principles [6, 18, 53, 60, 63, 69].

This skill evolution mirrors broader patterns observed across automated industries, where technological advancement creates opportunities for workers to engage in higher value, more intellectually demanding activities. The medical profession's trajectory follows this established pattern, with AI automation serving as a catalyst for professional advancement rather than replacement.

## EMBRACING THE PHYSICIAN'S EVOLUTION

### The current wave as an invaluable assistant

AI will serve as an invaluable assistant that enhances physician performance [43, 44]. Clinical studies reveal consistent improvements in diagnostic accuracy when physicians work alongside AI systems [39, 41, 42, 43, 44, 60]. Radiologists using AI assistance for COVID‐19 detection achieved almost perfect accuracy, while AI augmentation reduced clinical documentation time by 18%, maintaining diagnostic accuracy [8, 16].

A review of AI‐physician collaboration shows that human‐AI teams outperform either humans or AI systems working independently [23]. In polyp detection during colonoscopy, AI‐assisted endoscopists demonstrate superior adenoma detection rates, while AI‐augmented mammography reading reduces both false positives and missed diagnoses [3, 45, 64]. These findings highlight that the current generation of medical AI functions as a powerful amplifier of human expertise.

### The future wave: Logical and necessary skill evolution

As AI systems eventually achieve full automation in specific medical domains, the resulting transformation will represent a logical and necessary shift in physician expertise toward higher‐value activities [7, 11, 19, 26, 66]. The medical profession's trajectory follows this historical precedent, with AI serving as a catalyst for professional advancement, human‐AI collaboration and a shift to higher‐value activities will change the function of doctors, leading to advancement rather than the eradication of medical knowledge and skills [54].

### Final vision: Operating at higher, more impactful levels

The ultimate goal of AI integration in healthcare is not to preserve current medical practices against technological change, but to leverage AI capabilities to enable physicians to operate at higher, more impactful levels of care [35]. This transformation focuses on the irreplaceable human elements that define excellent medical practice.

The physician of the future will not be diminished by AI but rather elevated to focus on the most challenging, meaningful and uniquely human aspects of medical care. This represents an opportunity to rediscover the profession's core mission: combining knowledge with compassionate care to serve patients at their most vulnerable moments (Box 1). Technology can augment clinical capabilities, but humans will remain irreplaceable for now.

Fact BOX 1 Augmentation defines the current era of artificial intelligence in orthopaedics, where the goal is not to replace the surgeon but to elevate performance through human‐AI collaboration that outperforms either entity working in isolation.

Never‐skilling poses a greater long‐term threat to medical education than deskilling; it occurs when trainees rely on automation so early in their development that they fail to acquire foundational clinical reasoning and procedural competencies.

By automating repetitive administrative tasks and documentation, ambient AI liberates cognitive bandwidth, allowing physicians to ‘upskill’ by refocusing on complex decision‐making, procedural excellence and patient empathy.

The ‘calculator precedent’ offers a historical parallel for the physician's evolution, suggesting that automation of routine tasks will migrate professional skills toward higher‐order thinking and the validation of algorithmic outputs.

AI functions as a tool for micro‐learning at the point of care, reinforcing diagnostic accuracy by requiring residents and surgeons to actively review, confirm or override machine‐generated recommendations.

The role of the modern physician must shift from data processor to agent manager, necessitating new competencies in technical usage, medical decision‐making regarding tool application and the ethical validation of AI systems.

## AUTHOR CONTRIBUTIONS

All listed authors have contributed substantially to this work. Felix C. Oettl, James Pruneski and Balint Zsidai performed literature review. Felix C. Oettl, James Pruneski and Balint Zsidai performed primary manuscript preparation. Editing and final manuscript preparation were performed by Yinan Yu, David Fendrich, Michael T. Hirschmann, Thomas Tischer, Stefano Zaffagnini and Kristian Samuelsson. All authors read and approved the final manuscript.

## CONFLICT OF INTEREST STATEMENT

Kristian Samuelsson is a member of the Board of Directors of Getinge AB (Publ) and medtech advisor to Carl Bennet AB. The remaining authors declare no conflicts of interest.

## ETHICS STATEMENT

The authors have nothing to report.

## Data Availability

Data sharing is not applicable to this article as no datasets were generated or analysed during the current study.
